# TP53/miR-34a-associated signaling targets *SERPINE1* expression in human pancreatic cancer

**DOI:** 10.18632/aging.102776

**Published:** 2020-01-27

**Authors:** Shaw M. Akula, Peter P. Ruvolo, James A. McCubrey

**Affiliations:** 1 Department of Microbiology and Immunology, Brody School of Medicine, East Carolina University, Greenville, NC 27834,USA; 2 Department of Leukemia, University of Texas MD Anderson Cancer Center, Houston, TX 77030, USA

**Keywords:** PDAC, Aging, cancer, TP53, miR-34a, *SERPINE1*

## Abstract

Pancreatic ductal adenocarcinoma (PDAC) is a disease of aging. The *TP53* gene product regulates cell growth, aging, and cancer. To determine the important targets of TP53 in PDAC, we examined the expression of 440 proteins on a reverse phase protein array (RPPA) in PDAC-derived MIA-PaCa-2 cells which either had WT-*TP53* or lacked WT-*TP53*. MIA-PaCa-2 cells have a *TP53* mutation as well as mutant *KRAS* and represent a good *in vitro* model to study PDAC. RPPA analysis demonstrated expression of tumor promoting proteins in cells that lacked WT-*TP53*; and this feature could be reversed significantly when the cells were transfected with vector encoding WT-*TP53* or treated with berberine or a modified berberine (BBR). Expression of miR-34a-associated signaling was elevated in cells expressing WT-*TP53* compared to cells expressing *mTP53*. Results from *in vivo* studies using human PDAC specimens confirmed the *in vitro* results as the expression of miR-34a and associated signaling was significantly decreased in PDAC specimens compared to non-cancerous tissues. This study determined *SERPINE1* as a miR-34a target with relevance to the biology of PDAC. Thus, we have identified a key target (*SERPINE1*) of the TP53/miR-34a axis that may serve as a potential biomarker for early detection of pancreatic cancer.

## INTRODUCTION

The risk of developing cancer of the pancreas increases with age; it was estimated that only 13% of all patients with pancreatic cancer are diagnosed before the age of 60 [1]. The increasing incidence and mortality from pancreatic ductal adenocarcinoma (PDAC) are medical issues of paramount importance [2, 3]. Current treatments combining surgical resection and chemotherapy are only minimally effective [4, 5]. In most cases, by the time PDAC is diagnosed, it has already spread to distant sites, making treatment an impossible task. PDAC is the ninth most common cancer in the USA, has the highest mortality of any cancer, and will soon be the second most common cause of cancer death in USA [6, 7].

Two of the key genes involved in the development of PDAC are *KRAS* and *TP53* [8]. *KRAS* (activation) mutations occur in about 90% of PDAC while *TP53* (inactivation) mutations occur in approximately 75% of pancreatic cancers [9]. Apart from mutations in these genes, host cell microRNAs (miRNAs) also have crucial roles to play in various biological processes, including: inflammation, cell growth, aging, differentiation, proliferation, and metastasis [10, 11]. Increasing evidence in recent years suggests that miRNAs control the development and progression of inflammation and cancer [12–15]. In this study we focused on miR-34a over other miRNAs because of the following reasons: (i) Expression of miR-34a is significantly down-regulated or absent in a variety of cancers including hepatocellular and renal cell carcinomas, colon, breast, lung, prostate, ovarian, and pancreatic cancers [16–22]; (ii) The two major oncogenes that are mutated in PDAC are *KRAS* and *TP53* [23]; (iii) *TP53* directly transactivates miR-34a expression [24] while mutated *KRAS* indirectly lowers expression of miR-34a via the transcription factor, ZEB1 [25, 26]. Therefore, inactivation of *TP53* and increases in mutated *KRAS* expression result in a sharp decline in miR-34a expression during tumorigenesis.

The miR-34 family contains three members and is encoded by two genes located on chromosomes 1 and 11 [27]. The mature miR-34a shares 86% identity (19/22 nt) with miR-34b and 82% identity (18/22 nt) with miR-34c, respectively. The position 2-9 adjacent at the 5' end (8 nt) is considered the “seed region” for all three members [27–29]. Among these members, miR-34a is expressed at higher levels than miR-34b/c, with the exception of the lung [30].

miR-34a is a key regulator of tumor suppression and is considered to have a broad anti-oncogenic activity [30]. We hypothesize miR-34a to play a major role in the development of PDAC. As of this date, there are limited investigations conducted to understand the roles of miR-34a in the biology of PDAC. Therefore, the focus of this study was to decipher a potential role for TP53>miR-34a-associated signaling in pancreatic cancer using *in vitro* and *in vivo* models. Our study determined a decrease in the expression of miR-34a in human PDAC specimens. Using *in vitro* and *in vivo* approaches, we ascertained *SERPINE1* to be a target of miR-34a and their patho-physiological significance is discussed.

## RESULTS

### Profiling of tumor promoting and suppressor proteins in response to expression of wild-type TP53 in MIA-PaCa-2 cells

**Figure 1:**
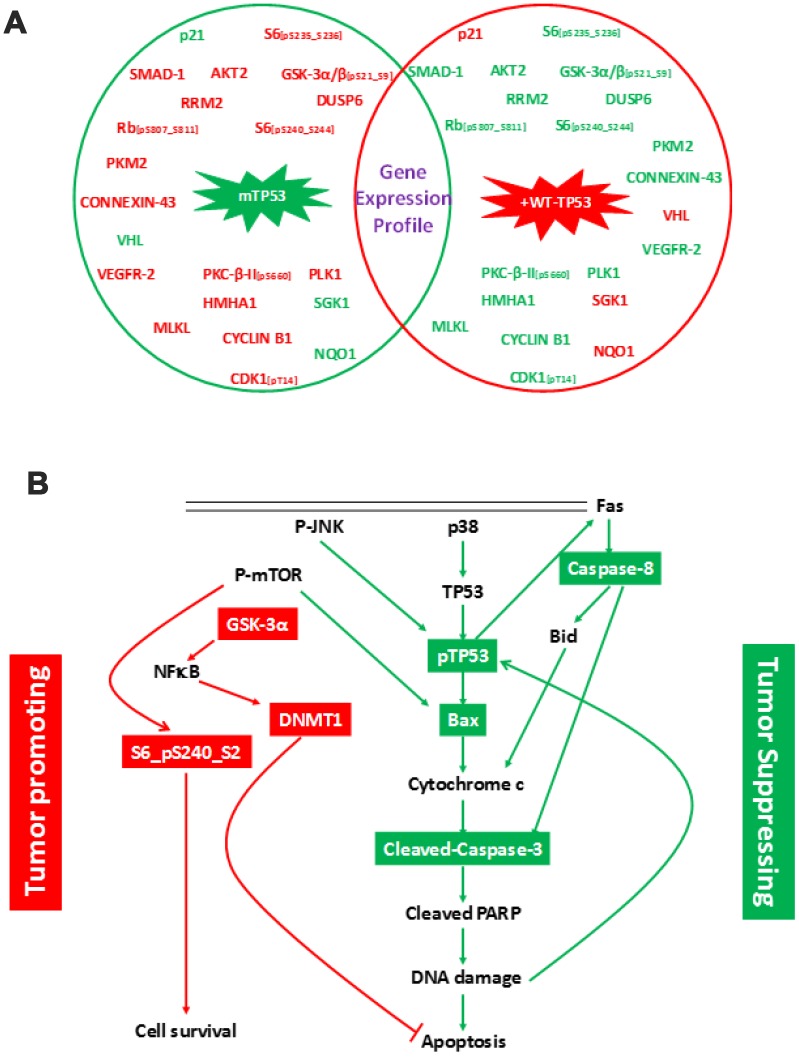
**Changes in protein expression profile in MIA-PaCa-2 cells expressing pLXSN compared to WT-TP53.** (**A**) Protein expression was assayed by RPPA. Proteins indicated in red and green denotes increased and decreased expression, respectively. Genes in red and green indicate tumor promoting and suppressor activities, respectively. (**B**) Schematic demonstrating cell signaling in MIA-PaCa-2+pLXSN cells promoting cell survival (in red) while significantly inhibiting apoptosis (in green).

RPPA assay was performed to elucidate the effects of expressing WT-*TP53* in MIA-PaCa-2 cells. The crucial step prior to performing the RPPA assay was to characterize the MIA-PaCa-2 cells used in this study. This is important as these cells expressing the *mTP53* and WT-*TP53* form the basis for the *in vitro* experiments conducted in this study. The MIA-PaCa-2+WT-*TP53* cells were more sensitive to the chemotherapeutic drugs compared to MIA-PaCa-2+pLXSN cells (Supplementary Figure 1). Similar results have been reported by earlier studies [23, 31–33]. The above results authenticate the physiological effects of expressing different forms of TP53 and associated cell signaling. RPPA is a high-throughput technology based on the detection of proteins along with their post-translational protein modifications, e.g., cleavage and phosphorylation [34]. To this end, we performed RPPA using a selection of 446 antibodies (Supplementary Table 1). RPPA analysis revealed a *mTP53*-dependent modulation of multiple cell signaling molecules involved in cell proliferation and survival (Figure 1A). Further, the analysis documented an increase and decrease in the expression of specific proteins that promoted tumor formation (Table 1) in MIA-PaCa-2 cells with mutated *TP53* (MIA-PaCa-2+pLXSN) compared to MIA-PaCa-2 cells expressing WT*-TP53* (MIA-PaCa-2+WT-*TP53*)*.* The expression of proteins in parental MIA-PaCa-2 untransfected cells followed a similar pattern as expressed in MIA-PaCa-2+pLXSN cells (data not shown).

**Table 1:** RPPA analysis demonstrating the tumor promoting milieu in MIA-PaCa-2+pLXSN cells compared to MIA-PaCa-2+WT-*TP53* cells.

**Protein name, and phosphorylation status**	**Gene symbol**	**Function**	**GenBank accession no.**	**Fold change in protein expression**
INCREASE IN EXPRESSION:				
AKT serine/threonine kinase 2 (AKT2)	AKT2	Promotes cancer formation	AAI20996.1	2.0
Cyclin dependent kinase 1 (CDK1_pT14))	CDK1	Promotes cell division	NP_001777.1	2.8
Connexin-43 (Cx43)	GJA1	Correlates with cancer metastasis	AAA52131.1	5.0
Cyclin-B1	CCNB1	Promotes cell survival	EAW51306.1	2.4
Dual specificity phosphatase 6 (DUSP6)	DUSP6	Drives poor prognosis in cancer	BAA34369.1	3.2
Glycogen synthase kinase 3α/β (GSK-3α/β_pS21_S9)	GSK-3α/β	Promotes cell growth & invasion	NP_063937.2	2.1
Minor histocompatibility protein HA-1 (HMHA1)	HMHA1	Induces cell spread	AAH48129.1	5.3
mitogen-activated protein kinase kinase kinase 9 (MLK1)	MLK1	Induces necroptosis	AAB26359.1	2.7
Protein kinase-β II (PKC-β-II_pS660)	PRKCB	Promotes signaling to cause cancer	P05771.4	2.0
Pyruvate kinase M1/2 (PKM2)	PKM2	Drives poor prognosis in cancer	AAH94767.1	2.1
Polo like kinase 1 (PLK1)	PLK1	Promotes proliferation and suppress apoptosis	NP_005021.2	3.1
Retinoblastoma protein (Rb_pS807_S811)	Rb1	Phosphorylation of Rb inactivates the protein	AAH40540.1	2.7
Ribonucleotide reductase regulatory subunit M2 (RRM2)	RRM2	Drives poor prognosis in cancer	NP_001025.1	2.4
40S ribosomal protein S6 (S6_pS235_S236)	S6	Promotes cell survival	NP_001001.2	3.4
40S ribosomal protein S6 (S6_pS240-S244)	S6	Promotes cell survival	NP_001001.2	3.8
SMAD family member 1 (SMAD1)	SMAD1	A crucial role in development of cancer	AAC50790.1	2.0
Vascular endothelial growth factor receptor-2 (VEGFR-2)	VEGFR-2	Induces angiogenesis	P35968.2	2.5
DECREASE IN EXPRESSION:				
NAD(P)H quinone dehydrogenase 1	NQO1	Regulates autophagy	AAI07740.1	0.3
p21	P21	Tumor suppressor	AAB29246.1	0.5
Serum/Glucocorticoid Regulated Kinase 1 (SGK1)	SGK1	Inhibits cancer cell invasion and migration	AAH01263.1	0.4
von Hippel-Lindau tumor suppressor (VHL)	VHL	Tumor suppressor	AAH58831.1	0.4

Expression of DNMT1, S6 (phosphorylated on serine residues at 240 and 244), and GSK-3α/3β (phosphorylated on serine residue at 21 of GSK3α or serine 9 of GSK-3β) were elevated in MIA-PaCa-2 cells with *mTP53* (MIA-PaCa-2+pLXSN) (Table 2) and MIA-PaCa-2 cells (data not shown). On the same lines, expression of Bax, cleaved caspase-3, and cleaved caspase-8 were down-regulated in MIA-PaCa-2 cells expressing WT-*TP53* (MIA-PaCa-2+WT-*TP53*) (Table 2). Thus, the cellular events seem to promote cell survival while actually inhibiting apoptosis in cells expressing *mTP53* (Figure 1B). RPPA analysis demonstrated a crucial role for the WT-*TP53* in mediating anti-tumor activity via modulating cell signaling.

**Table 2:** RPPA analysis demonstrating changes in the expression of proteins that promote cell survival while decreasing apoptosis in MIA-PaCa-2+pLXSN cells compared to MIA-PaCa-2+WT-TP53 cells.

**Protein name, and phosphorylation status**	**Gene symbol**	**Function**	**GenBank accession no.**	**Fold change in protein expression**
PROMOTING CELL SURVIVAL:				
DNA methyltransferase 1 (DNMT1)	DNMT1	Promotes cell survival	AAI26228.1	2.4
40S ribosomal protein S6 (S6_pS240-S244)	S6	Promotes cell survival	NP_001001.2	3.8
Glycogen synthase kinase 3α/β (GSK-3α/β_pS21_S9)	GSK-3α/β	Promotes cell survival	NP_063937.2	2.1
DECREASING TUMOR SUPPRESSION:				
BCL2 associated X, apoptosis regulator (BAX)	BAX	Promotes apoptosis	Q07812.1	0.3
Cleaved caspase-3 (Caspase-3)	CASP3	Promotes apoptosis	CAC88866.1	0.4
Cleaved caspase-8 (Caspase-8)	CASP8	Promotes apoptosis	BAB32555.1	0.3

### Effect of treating MIA-PaCa-2 cells with BBR and MBBR on cell division, proliferation, survival, migration, and apoptosis

**Figure 2:**
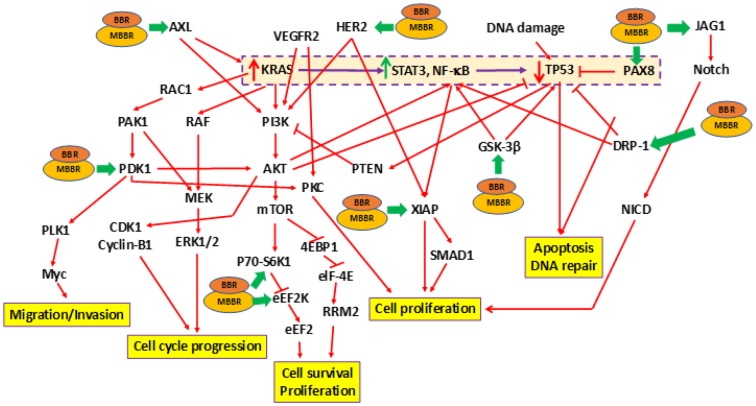
**Effects of treating MIA-PaCa-2 cells with BBR and NAX060 on cell division, proliferation, survival, migration, and apoptosis.** A schematic depicting the effects of BBR and NAX060 on the *N-RAS*/*TP53*-associated signaling critical to PDAC development. The model is based on the fact that over-expression of mutated *KRAS* significantly enhances STAT3, NF-κB signaling which in turn lowers the TP53 expression (highlighted and boxed in dotted purple line). Green bold arrows denote inhibiting effects of BBR/MBBR on the signaling molecule.

Earlier studies by us determined that BBR and MBBR inhibited proliferation of pancreatic cancer cells [31, 32]. In the current study, we determined the effect of treating MIA-PaCa-2+pLXSN cells with BBR and MBBR (NAX060) on cell signaling using RPPA. Treatment of MIA-PaCa-2+pLXSN cells (carrying *mTP3*) with BBR and MBBR altered the expression of 11 proteins to varying extents (Table 3). Each of these proteins influence tumorigenesis by regulating cell cycle progression, survival, proliferation, apoptosis and DNA repair. The effects of BBR and MBBR on the proliferation of MIA-PaCa-2+pLXSN cells is presented in the schematic (Figure 2). The schematic also represents the manner by which BBR and MBBR may directly or indirectly alter the expression of *mTP53*-associated signaling molecules (Figure 1A). RPPA analysis demonstrated the ability of BBR and MBBR to promote anti-tumor activity in MIA-PaCa-2+pLXSN and MIA-PaCa-2 (data not shown) cells by inhibiting cell cycle progression, proliferation, and survival to varying extents.

**Table 3:** RPPA analysis demonstrating the fold change in activity of proteins in response to treating MIA-PaCa-2+pLXSN cells with BBR and MBBR.

**Protein name, and phosphorylation status**	**Gene symbol**	**Function**	**GenBank accession no.**	**% drop in expression**	
				**BBR**	**MBBR**
AXL receptor tyrosine kinase (AXL)	AXL	Promotes proliferation, stem cell phenotype	AAH32229.1	34%	46%
Dynamin-related protein 1 (DRP-1)	DRP-1	Promotes cell survival, migration	O00429.2	44%	43%
Eukaryotic elongation factor 2 kinase (eEf2K)	eEf2K	Promotes cell survival, proliferation	AAH32665.1	31%	38%
Glycogen synthase kinase 3α/β (GSK-3α/β_pS21_S9)	GSK-3α/β	Promotes cell growth & invasion	NP_063937.2	92%	33%
Human epidermal growth factor receptor 2 (HER2)	HER2	Correlates with worse survival	P04626.1	39%	92%
Jagged canonical Notch ligand 1 (JAG1)	JAG1	Promotes migration and invasion of cells	NP_000205.1	38%	42%
Paired box 8 (PAX8)	PAX8	Promotes cell proliferation	AAB34216.1	54%	44%
Pyruvate dehydrogenase kinase 1 (PDK1)	PDK1	Promotes cell growth and survival	AAH39158.1	86%	35%
Ribosomal protein S6 kinase B1 (S6K1)	S6K1	Promotes cell proliferation	P23443.2	52%	37%
X-linked inhibitor of apoptosis (XIAP)	XIAP	Inhibitor of apoptosis	NP_001191330.1	70%	33%

### WT-TP53 enhances expression of miR-34a in MIA-PaCa2 cells

**Figure 3:**
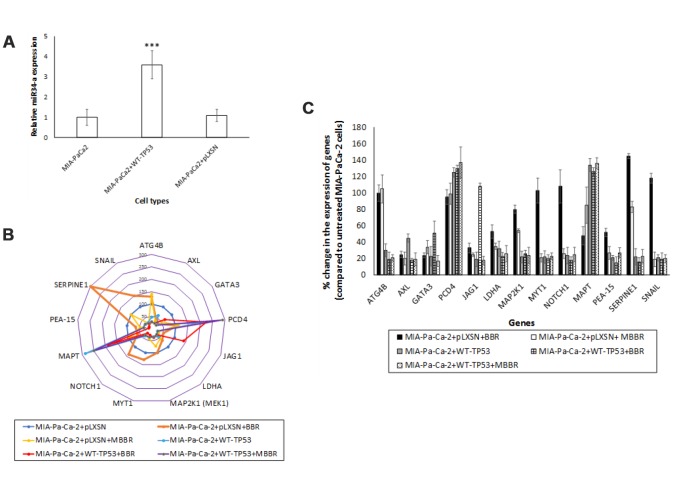
**miR-34a expression in MIA-PaCa-2+pLXSN cells.** (**A**) qRT-PCR was conducted to determine the miR-34a expression in MIA-PaCa-2+WT-TP53 and MIA-PaCa-2+pLXSN cells. Briefly, approximately 500 ng of RNA was reverse transcribed in a 25 μl reaction volume using the All-in-one miRNA qRT-PCR detection kit (GeneCopoeia, Rockville, MD). The synthesized cDNAs were used in the PCR reaction. The expression levels of miR-34a were measured employing the SYBR green detection and specific forward primer for the mature miRNA sequence and the universal adaptor reverse primer (GeneCopoeia, USA). Two-tailed P value of 0.05 or less was considered statistically significant; ***p < 0.001. (**B**) The putative targets of miR-34a that were significantly altered in MIA-PaCa-2+pLXSN and MIA-PaCa-2+WT-TP53 cells when the cells were treated with BBR and MBBR. A select few of the miR-34a target proteins that were significantly altered by treatment of MIA-PaCa-2 cells with BBR and NAX060 are projected. The data represent average of three individual experiments. (**C**) qRT-PCR was conducted to determine the expression of miR-34a-target genes in MIA-PaCa2+pLXSN and MIA-PaCa-2+WT-*TP53* cells. qRT-PCR was performed to monitor expression of the different miR-34a-putative target genes in untreated MIA-PaCa-2+pLXSN cells and MIA-PaCa-2 expressing WT-*TP53* or those treated with BBR and MBBR, respectively, using specific primers and SYBR green detection as per standard protocols. Bars represent average ± s.d. of three individual experiments.

TP53 directly transactivates miR-34a expression [24]. Therefore, we set out to compare the expression levels of miR-34a in MIA-PaCa-2 cells *in vitro*. The expression levels of miR-34a were significantly lower in the pancreatic cancer cell lines MIA-PaCa-2 and MIA-PaCa-2+pLXSN than those of MIA-PaCa2 cells that were stably transfected with vector encoding WT-*TP53* (MIA-PaCa-2+WT-*TP53*) (Figure 3A). Mock transfection (data not shown) did not significantly alter the expression profile of miR-34a. These results indicate the following: a) miR-34a levels are inherently lower in cells derived from pancreatic cancer which have a *mTP53*; and b) There is a direct positive correlation between the expression of WT-*TP53* and miR-34a.

One miRNA may target several genes. By using the miRmap and PiCTar tool algorithms [35, 36], we identified potential targets for miR-34a (Supplementary Tables 2 and 3). Analysis of RPPA data identified expression of a few of the miR-34a target proteins was altered in MIA-PaCa-2 cells. We determined a significant decrease in the expression of putative miR-34a targets (*ATG4B, AXL, GATA3, JAG1, LDHA, MAP2K1, MYT1, NOTCH1, PEA-15, SERPINE1*, and *SNAIL*) in MIA-PaCa-2+WT-*TP53* compared to MIA-PaCa-2+pLXSN (Figure 3B). Expression of putative miR-34a targets (PCD4 and MAPT) were significantly elevated in MIA-PaCa-2+WT-*TP53* compared to MIA-PaCa-2+pLXSN (Figure 3B). The effect of expressing WT-*TP53* on the miR-34a targets at the level of transcription was monitored in cells by qRT-PCR. qRT-PCR data (Figure 3C) corroborated the RPPA analysis. The study established an inverse correlation between the expression of miR-34a and its target genes.

### *In vivo* expression profile of miR-34a reflects its *in vitro* expression pattern

**Figure 4:**
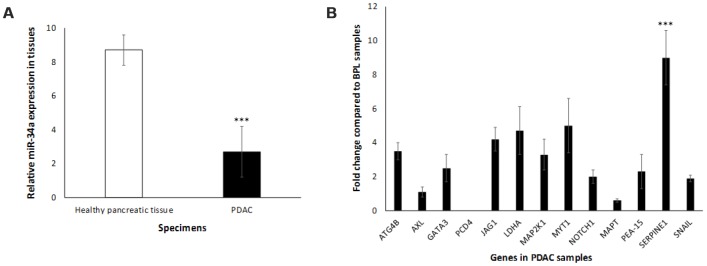
**Expression profile of miR-34a in human PDAC samples.** (**A**) miR34-a expression levels are lower in PDAC specimens compared to healthy pancreas controls. We compared the expression of miR-34a in 10 specimens in each group. Student t test was performed to compare groups. Two-tailed *P* value of 0.05 or less was considered statistically significant. ***p < 0.001. (**B**) qRT-PCR was conducted to determine the expression of miR-34a-target genes in human PDAC or healthy pancreas control specimens. Expression of miR-34a-target genes in human PDAC and healthy pancreas control specimens were detected by qRT-PCR using specific primers and SYBR green detection as per standard protocols. Bars represent average ± s.d. of three individual experiments. Two-tailed P value of 0.05 or less was considered statistically significant; ***p < 0.001.

To monitor *in vivo* expression of miR-34a, we used human pancreas samples obtained from PDAC patients with appropriate controls. The expression levels of miR-34a were measured employing qRT-PCR with the SYBR green detection and specific forward primer for the mature miRNA sequence [74] and the universal adaptor reverse primer (GeneCopoeia, USA). Our preliminary results (Figure 4A) demonstrate a significant decrease in the levels of miR-34a in PDAC tumors when compared to healthy pancreas controls. The next obvious question was to understand the expression profiles of the set of putative miR-34a target genes that were significantly altered *in vitro* (Figure 3B, 3C). The expression profile of the miR-34a target genes (*ATG4B, AXL, GATA3, JAG1, LDHA, MAP2K1, MYT1, NOTCH1, PEA-15, SERPINE1,* and *SNAIL*) followed an identical expression pattern (Figure 4B). Expression of *PCD4* was at undetectable levels *in vivo* (Figure 4B). Interestingly, expression of *SERPINE1* was significantly greater than any other miR-34a target genes of interest. This along with the fact that little is known about miR-34a>SERPINE1 associated signaling led us to further investigate the biology of this interaction in pancreatic cancer.

### miR-34a targets *SERPINE1*

**Figure 5:**
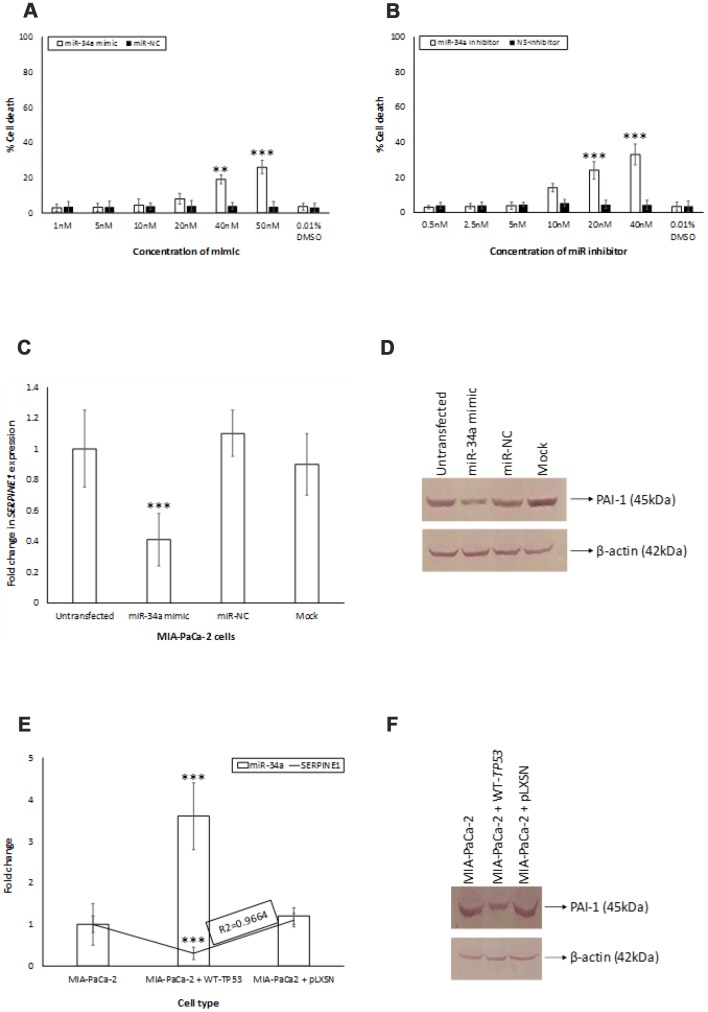
**miR-34a targets *SERPINE1*.** (**A**, **B**) To determine the cytotoxic effect of miR-34a mimic and inhibitor, MIA-PaCa2+pLXSN cells were transfected with different concentrations of miR-34a mimic and inhibitor. At 24 h post transfection, MTT was added to each well and the absorption was measured. Percentage of cell death was monitored for miR-34a mimic (miR-mimic) (**A**) and miR-inhibitor (**B**) compared with 0.01% DMSO as control. (**C**, **D**) miR-34a mimic significantly decreased expression of *SERPINE1* and PAI-1 in MIA-PaCa-2+pLXSN cells. MIA-PaCa-2+pLXSN cells were untransfected, mock transfected, or transfected with miR-34a mimic or miR-NC. At the end of 24h of incubation at 37°C, the cells were lysed, RNA extracted (panel **C**), cDNA synthesized, and *SERPINE1* expression monitored by qRT-PCR. In another set of experiments, the cells were lysed were probed for PAI-1 expression by Western blotting (panel **D**). (**E**) The relative expression of *SERPINE1* and miR-34a in MIA-PaCa-2 target cells was monitored by qRT-PCR. The expression was measured in terms of cycle threshold value (Ct) and normalized to expression of β-actin and snRNA RNU6B, respectively. The x-axis denotes the cell type and y-axis denotes fold change in expression of *SERPINE1* and miR-34a. The R2 values for the miRNA expression are provided. (**F**) In another set of experiments, the above cells were lysed and probed for PAI-1 expression by Western blotting (panel **F**). Bars (**A**–**C**, **E**) represent average ± s.d. of five individual experiments. Student t test was performed to compare groups. Two-tailed P value of 0.05 or less was considered statistically significant. **p,0.01; ***p < 0.001; NS-not significant.

The secondary structure of the pre-miR-34a was predicted using the RNAstructure software [37] (Supplementary Figure 2). By using the DIANA and MiRmap tool algorithms, we identified a putative miR-34a binding site located in the 3′-UTR of *SERPINE1* mRNA (Supplementary Figure 3). To confirm the ability of miR-34a to specifically inhibit *SERPINE1* expression, we monitored the expression of *SERPINE1* in target cells that were untransfected, transfected with miR-34A mimic, or miR-NC. The range of doses tested in this study is comparable to those reported in the earlier studies [38–40]. The doses of the mimic and inhibitor used in the study did not significantly induce cell death in MIApaCa-2+pLXSN cells (Figure 5A, 5B). Transfection of MIA-PaCa-2+pLXSN cells with the miR-34a mimic significantly lowered the expression of *SERPINE1* and *SERPINE1* encoded protein, plasminogen activator inhibitor (PAI-1) levels at 24h post transfection compared to untransfected cells and cells transfected with miR-NC (Figure 5C, 5D). There was an inverse correlation observed in the expression of miR-34a and *SERPINE1* and PAI-1 levels in MIA-PaCa-2+pLXSN cells (Figure 5D, 5E). These results authenticate the fact that *SERPINE1* expression may well be regulated by miR-34a.

**Figure 6:**
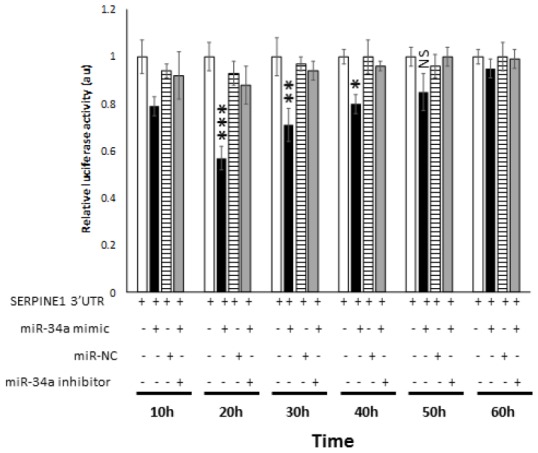
**miR-34a specifically binds and interact with *SERPINE1*.** Luciferase activity in 293 cells transfected with Dual-luciferase vector encoding Gaussia Luciferase (GLuc) and secreted alkaline phosphatase (SEAP) with 3′UR of *SERPINE1* placed downstream of Glu luciferase reporter (*SERPINE1* 3′UTR). 293 cells were either transfected with *SERPINE1* 3′UTR, co-transfected with *SERPINE1* 3′UTR and miR-34a mimic, co-transfected with *SERPINE1* 3′UTR and control mimic (miR-NC), or co-transfected with *SERPINE1* 3′UTR, miR-34a mimic and miR-34a inhibitor. GLuc activity was monitored at 10 h, 22 h, 30 h, 40h, 50 h, and 60 h post-transfection and was normalized to SEAP. Data is plotted as GLuc/SEAP ratio where the x-axis indicates the transfection and time points, and y-axis indicates the relative luciferase activity. Bars represent average ± s.d. of five individual experiments. Student t test was performed to compare groups. Two-tailed P value of 0.05 or less was considered statistically significant. *p < 0.05; **p,0.01; ***p < 0.001; NS-not significant.

In order to determine the *bona fide* target of miR-34a, a luciferase reporter assay was performed. In this assay, two quantifiable genes encoding luciferase proteins were cloned in a vector. The *SERPINE1* 3′ UTR with the target region was placed downstream GLuc to regulate its translation, and SEAP was placed under no regulation for normalization. 293 cells were co-transfected with the *SERPINE1* 3′ UTR vector plasmid and miR-34a mimic. miR-34a mimic significantly decreased the relative luciferase activity compared to the cells that were transfected with miR-NC (Figure 6). In contrast, transfection of cells with miR-inhibitor reversed the ability of miR-34a mimic from lowering the luciferase activity (Figure 6). These results suggest that miR-34a directly targets *SERPINE1* and thereby downregulates its expression.

## DISCUSSION

The *TP53* tumor suppressor gene is also known as the “guardian of the genome” as it serves to identify DNA damage, pause cell cycle progression to allow for repair, and when repair is not possible, to induce apoptosis [41, 42]. The multiplatform molecular analysis of the PDAC-derived target cells exhibits a range of neoplastic cellularity representative of the clinico-pathologic spectrum of this disease (Figure 1). The RPPA analysis demonstrated the following: (i) expression of *mTP53*-associated signaling promoted cell survival and proliferation while inhibiting apoptosis (Figure 1; Tables 1, 2). Cells with *mTP53* alone (MIA-PaCa-2+pLXSN) had an increase in the expression DUSP6 (Figure 1). The role of DUSP6 in tumor formation depends on the micro-environment [43]. Recent studies demonstrated over-expression of DUSP6 to induce tumor formation [44]; (ii) expression of WT-*TP53* had an opposing effect on *mTP53*-associated signaling (Figure 1); (iii) Treatment of cells expressing *mTP53* with BBR and MBBR can reverse cell signaling critical to tumor formation (Figure 2; Table 3). Inactivation of *TP53* is believed to be a critical step in pancreatic cancer progression. The above results are a crucial piece of evidence to this work on miRNA as this allowed us to establish a cell culture model to study the effects of TP53 on miR-34a and associated signaling.

*TP53* mutations frequently occur during the transition from benign pancreatic intra-epithelial neoplasia to the highly-aggressive, invasive and metastatic PDAC [45]. TP53 is a transcription factor that controls the expression of many key genes and miRNAs that are involved in the regulation of cell cycle progression, apoptosis, cellular senescence and other critical biological processes [46–48]. miR-34a expression in PDAC-derived cell lines like MIA-PaCa-2 cells is relatively low [49]. It was demonstrated in this study that miR-34a levels could be significantly increased in the same MIA-PaCa-2 cells when they were transfected with vector expressing WT-*TP53* (Figure 3A). RPPA analysis also demonstrated a sharp decline in the expression of miR-34a-associated target genes in MIA-PaCa-2 cells over-expressing WT-*TP53* compared to cells expressing *mTP53* (Figure 3B, 3C). Overall, this is the first report to demonstrate a direct correlation between the WT-*TP53* and miR-34a expression in PDAC-derived cells.

In order to appreciate the clinical relevance of the expression of miR-34a and its cognate targets *in vivo*, we monitored the expression profiles of miR-34a and associated signaling *in vivo* using PDAC specimens derived from human participants. miR-34a levels were significantly lower in PDAC specimens compared to healthy pancreatic tissues (Figure 4A). Also, we observed an increase in the expression of majority of the miR-34a targets (Figure 4B) that were analyzed by RPPA using lysates from MIA-PaCa-2 cells (Figure 3B, 3C). The only difference observed was as follows: (i) *in vivo* expression of *PCD4* was at undetectable levels; and (ii) expression of *SERPINE1* was significantly elevated compared to the rest of the miR-34a targets (Figure 5B). *SERPINE1* levels have been identified to be significantly increased in colorectal cancer [50], lung cancer [51], gastric cancer [52], bladder cancer [53], head and neck squamous cell carcinoma [54], and others. Interestingly, earlier studies demonstrated ability of the *SERPINE1* encoded protein, plasminogen activator inhibitor (PAI-1), to mediate proliferation and invasion of PDAC-derived cell lines, including MIA-PaCa-2 cells [55]. A recent study also concluded that the expression of *SERPINE1* is negatively-related to the survival of PDAC patients [56]. Nonetheless, there are only three manuscripts that describe the expression of *SERPINE1* and its association with PDAC and they were all performed with cell line models [55–57]. This is the first report of that links miR-34a>*SERPINE1* expressions to PDAC using an *in vivo* patient-derived sample model.

It is a known fact that multiple genes may be regulated by one miRNA [58]. On the same note, a single mRNA transcript may be regulated by multiple miRNAs [59]. It is more than likely that the relationships between miRNAs and their targets are not one-to-one but multiple-to-multiple in cancers as reported in gastric carcinogenesis [60]. Earlier studies have demonstrated *SERPINE1* as a target of miR-34a in colorectal [61] and non-small cell lung cancer [62]. Using bioinformatics tools, we identified *SERPINE1* to be a promising target to miR-34a (Supplementary Figure 3). The results from luciferase reporter assays confirmed *SERPINE1* to be a target for miR-34a (Figure 6). Accordingly, there was an inverse correlation between the expression of miR-34a and *SERPINE1* (Figure 5E). Taken together, our results for the first time demonstrates a direct link between TP53, miR-34a, and SERPINE1 expression profiles in the pathobiology of PDAC.

The *SERPINE1* gene is located at 7q21.2-q22 and encodes a single-chain glycoprotein of about 50kDa. The *SERPINE1* gene is one of the main regulators of the plasminogen activator system (PAs). SERPINE1 inhibits the urokinase-type plasminogen (uPA) and tissue-type plasminogen activator (tPA), which in turn, reduce the conversion of plasminogen to the active protease plasmin [21]. Thus, the plasminogen activator inhibitor-1 (PAI-1) encoded by the *SERPINE1* gene regulates tumor cell migration and invasion crucial to tissue remodeling and tumorigenesis [63, 64]. PAI-1 protein can exist in two distinct forms: active and inactive forms. This is crucial because depending on the conformation, PAI-1 can activate distinct cell signaling pathways critical to development of tumors [65].

miR-34a expression inhibits components of inflammatory response [66]. miR-34a downregulates expression of NF-κB via APE1/Ref-1 or SEMA4B [67, 68]. Importantly, miR-34a targets more TP53 network genes compared to miR-34b/c [24]. miR-34a is a key regulator of tumor suppression and is considered to have a broad anti-oncogenic activity [30]. Expression of miR-34a is significantly down-regulated or absent in a variety of cancers including hepatocellular and renal cell carcinomas, colon, breast, lung, prostate, ovarian, and pancreatic cancers [16–22]. The focus of this study was on miR-34a; which is the target of TP53 [69]. In the process, we were able to identify a key link between miR-34a, *SERPINE1*, and PDAC. Just as the age is a risk factor for the development of PDAC [70], PAI-1 is a part of the senescence-associated secretory phenotype (SASP) [71] and its expression is accordingly elevated in the elderly [72, 73]. Future studies are aimed at delineating the interactions between miR-34a and *SERPINE1* in the context of PDAC and aging.

## MATERIALS AND METHODS

### Cells

The MIA-PaCa-2 (ATCC® CRM-CRL-1420™) carcinoma cell line was derived from a 65-year old Caucasian male [74]. MIA-PaCa-2 cells have the R248W *TP53* GOF mutation. The R248W *TP53* mutation present in MIA-PaCa-2 cells is a missense point mutation in the central DNA binding domain which abrogates its DNA contact [75]. This *TP53* mutation results in a TP53 protein that is unable to bind to all TP53 target sequences in TP53-responsive genes and 2results in loss of its tumor suppressor properties [76, 77]. MIA-PaCa-2 cells also have an activating mutation at *KRAS* (G12C) and an elevated PI3K/AKT pathway activity. MIA-PaCa-2 cells were purchased from the ATCC (Rockville, MD, USA). Cells were cultured in medium containing 5% fetal bovine serum (FBS) purchased from (Atlanta Biologicals, Atlanta, GA, USA) as described in [33]. Tissue culture medium (Dulbecco's modified Eagles medium, DMEM), antibiotics containing l-glutamine and trypsin were obtained from Invitrogen (Carlsbad, CA, USA).

### BBR and modified BBR (NAX060)

BBR was purchased from Sigma-Aldrich (Saint Louis, MO, USA). NAX060 compound was synthesized, purified and provided as a gift by Dr. Paolo Lombardi (Naxospharma, Milan, Italy) [78, 79].

### Infection of cells with a retroviral vector encoding WT-*TP53*

The MIA-PaCa-2 cell line was infected with either a retroviral vector encoding WT-*TP53* (MIA-PaCa-2+WT-*TP53*) or the empty pLXSN vector (MIA-PaCa-2+pLXSN) as a control as described [23]. Stably infected cell lines were isolated in the presence of 2 mg/ml G418 (geneticin; Sigma-Aldrich). Pools were established after approximately four weeks in culture as per standard protocols [31].

### Reverse phase protein array (RPPA)

Target cells were either untreated or treated with 1,000 nM BBR or 1,000 nM NAX060 for 24h at 37°C. Cells were lysed 24 h later, denatured with 1% SDS and beta-mercaptoethanol, and five 2-fold serial dilutions of the samples were arrayed on nitrocellulose-coated slides (Grace Bio Lab, Bend, OR, USA) by an Aushon 2470 Arrayer (Aushon BioSystems, Bellerica, MA, USA). Each slide was probed with 419 primary antibodies and a biotin-conjugated secondary antibody. The stained samples were precipitated with 3,3' diaminobenzidine tetrahydrochloride (DAB) and quantified for spot intensity by using customized software. The signals were amplified with a Catalyzed Signal Amplification System (DakoCytomation, Glostrup, Denmark). Only target antibodies with a Pearson correlation coefficient (RPPA: western blotting) greater than 0.7 were used in the RPPA analysis. Each dilution curve was fitted with a logistic model (“Supercurve Fitting,” developed by the Department of Bioinformatics and Computational Biology at MD Anderson Cancer Center). R software and the package Ggplot2 were used to visualize the heatmap.

### Human PDAC specimens

A total of ten frozen PDAC human specimens were used in this study. We also used a total of ten frozen healthy pancreas specimens as controls. A total of these 20 samples were obtained from the North Carolina Tissue Consortium, Division of Surgical Oncology, Brody Medical Sciences Building, Greenville, NC. All these specimens were preserved in a liquid nitrogen container.

### Monitoring expression of miR-34a

RNA was extracted from the cells and the tissues as per standard laboratory procedures using TRIzol (Invitrogen) [38]. The RNA concentrations were measured with a NanoDrop ND-2000 spectrophotometer (Thermo Fisher Scientific, Waltham, MA, USA), and then verified for quality using an Agilent 2100 Bioanalyzer (Agilent Technologies, Santa Clara, CA, USA). Only the RNA samples with 260/280 ratios of 1.8 to 2.0 were used in the study.

Approximately 500 ng of RNA was reverse transcribed in a 25 μl reaction volume using the All-in-oneTM miRNA qRT-PCR detection kit (GeneCopoeia, Rockville, MD, USA). Briefly, the cDNA was synthesized in a 25 μl reaction mix containing 5 μl of 5x reaction buffer, 2.5U/μl poly A polymerase, 10ng/μl MS2 RNA, and 1μl RTase mix. The reaction was performed at 37°C for 60 min and terminated at 85°C for 5 min. cDNA that was produced in the RT reaction was diluted ten-fold and was used as the template for the PCR reaction in an Applied Biosystems ViiA 7 Real-Time PCR System (Thermo Fisher Scientific). In this system, MS2 RNA was used as an external reference for the quality of the extracted miRNAs, and RNU6B, RNU44, RNU48, and RNU49 were used for normalization. The expression levels of miRNAs were measured employing qRT-PCR with the SYBR green detection and specific forward primer for the mature miRNA sequence and the universal adaptor reverse primer (GeneCopoeia, USA). The specific forward primer to amplify miR-34a was 5’-TGGCAGTGTCTTAGCTGGTTGT-3’.

### qRT-PCR to monitor expression of miR-34a putative targets

RNA was extracted from the cells and the tissues as per standard laboratory procedures using TRIzol [38]. Expression of *ATG4B, AXL, GATA3, JAG1, LDHA, MAP2K1, MYT1, NOTCH1, PEA-15, SERPINE1*, and *SNAIL* mRNAs by qRT-PCR was conducted as per earlier protocols [58] using appropriate primers (Supplementary Table 4).

### Cytotoxicity assay

The 3-(4,5-dimethylthiazol-2-yl)-2,5-diphenyl-tetrazolium bromide (MTT) assays were performed to assess the sensitivity of cells to drugs, as previously described [23, 31, 80]. Target cells were treated with different concentrations of miR-34a mimic, inhibitor, or with appropriate controls at 37°C in a V-bottom 96-well plate. After a 24 h incubation, the percentage viable cells were assayed with MTT (Sigma-Aldrich). The optical density (OD) at the wavelength of 570 nm was used to calculate cell viability.

### Western blotting

All the buffers used in this project were made with water that was endotoxin and pyrogen free. Western blotting was conducted as per earlier studies using the following primary antibodies: rabbit anti-PAI-1 polyclonal antibody (ThermoFisher Scientific) and mouse anti-actin antibodies (Clone AC-74; Sigma-Aldridge).

### Dual-luciferase reporter assay

Luciferase reporter plasmids with wild-type *SERPINE1* 3′-UTR were purchased from GeneCopoeia. 293 cells were plated in 6-well plates. At 24 h post-plating, 293 cells were co-transfected with *SERPINE1* 3′-UTR luciferase reporter plasmid and miR-34a mimic, a scramble control (miR-NC), and/or miR-34a inhibitor using FuGene HD (Promega, Madison, WI, USA). At 10, 20, 30, 40, 50, and 60 h post transfection, supernatants were collected from each treatment and the luciferase activity measured using the Secrete-Pair Dual Luminescence Assay Kit (GeneCopoeia) as per the manufacturers’ recommendations.

## Supplementary Material

Supplementary Figures

Supplementary Table 1

Supplementary Table 2

Supplementary Table 3

Supplementary Table 4
