# Supplementary material for: TP53/miR-34a-associated signaling targets SERPINE1 expression in human pancreatic cancer
Source: Aging (Albany NY). 2020 Jan 27;12(3):2777–97. doi: 10.18632/aging.102776 (PMC7041729; doi:10.18632/aging.102776)
Supplement: Supplementary Figures [file aging-12-102776-s005..pdf]

SUPPLEMENTARY FIGURES

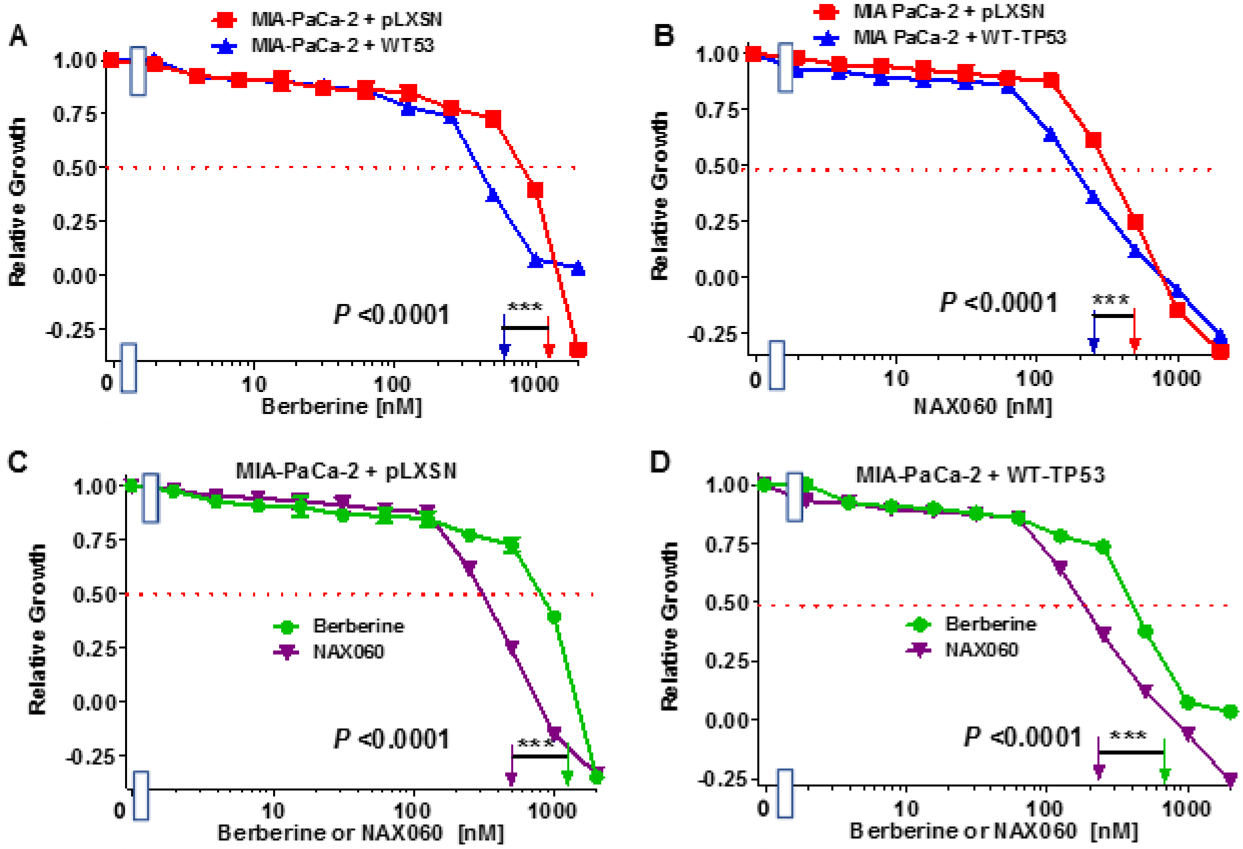

Supplementary Figure 1. Effects of Different doses of BBR and MBBR (NAX060) on MIA-PaCa-2+pLXSN and MIA-PaCa-2+WT-*TP53* Cells.

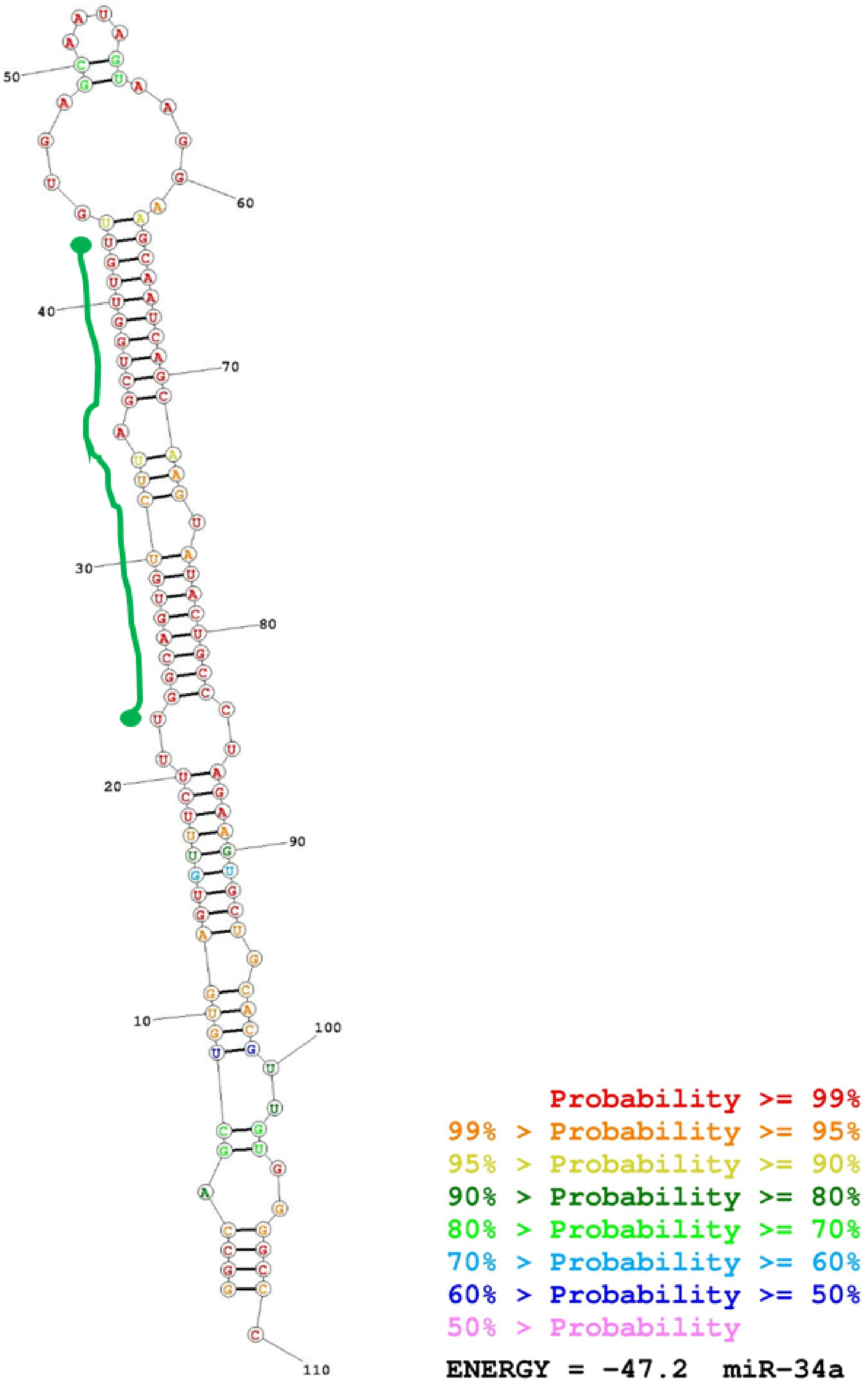

Supplementary Figure 2. Secondary structures of Pre-mir-34a. Structure was predicted using the RNAstructure software and base-pairing probability depicted in colors. Green line along the sequence denotes the mature sequence of hsa-miR-34a-5p (22 – 43).

```
 (SERPINE1      5' CAU       GUGACG    CCC             3'
Transcript)              CCAGC        GAG     ACACUGCCA
                         |||||        .||     |||||||||
                         GGUCG        UUC     UGUGACGGU
 (miR-34a)      3'   U       A                         5'
```

Transcript position: 940-965
Binding type: 9mer
Conserved species: panTro2,rheMac2,oryCun2,bosTau4,canFam2,dasNov2,loxAfr3,echTel1
miTG score: 0.906

Supplementary Figure 3. RNA hybrid analysis shows the miR-34a binding site located in 3'UTR of SERPINE1 mRNA. This is predicted using DIANA and MiRmap algorithms.
