# Supplementary material for: TP53/miR-34a-associated signaling targets SERPINE1 expression in human pancreatic cancer
Source: Aging (Albany NY). 2020 Jan 27;12(3):2777–97. doi: 10.18632/aging.102776 (PMC7041729; doi:10.18632/aging.102776)
Supplement: Supplementary Table 4 [file aging-12-102776-s004..pdf]

SUPPLEMENTARY TABLES

Please browse Full Text version to see the data of Supplementary Tables 1–3

Supplementary Table 1. Description of antibodies used in RPPA assay.

Supplementary Table 2. comprehensive list of the putative targets of miR-34a as determined by miRmap.

Supplementary Table 3. comprehensive list of the putative targets of miR-34a as determined by PicTar.

Supplementary Table 4. Primers used to amplify miR-34a putative targets.

Target	Primer
ATG4B	F: 5’-TGAGTCTTGTGGTGTGTGGT-3’
	R: 5’-TACTTTCCCAGGACAGGCAG-3’
AXL	F: 5’-GAGGGAGAGTTTGGAGCTGT-3’
	R: 5’-GAAACAGACACCGATGAGCC-3’
GATA3	F: 5’-GGCGCCGTCTTGATACTTTC-3’
	R: 5’-AAGAGCAGAGAGGAGGAGGA-3’
PCD4	F: 5’-GCAGAAAATGCTGGGACTGAG-3’
	R: 5’-TGTACCCCAGACACCTTTGC-3’
JAG1	F: 5’-GTCCCACTGGTTTCTCTGGA-3’
	R: 5’-ATATACCGCACCCCTTCAGG-3’
LDHA	F: 5’-GGCTACACATCCTGGGCTAT-3’
	R: 5’-TCTTCTTCAAACGGGCCTCT-3’
MAP2K1	F: 5’-CAGAAGCAGAAGGTGGGAGA-3’
	R: 5’-GGATTGCGGGTTTGATCTCC-3’
MYT1	F: 5’-TTGATGTCAAGCCTGCCAAC-3’
	R: 5’-CAGACTGAACACATCCGCTG-3’
NOTCH1	F: 5’-ATGCAGAACAACAGGGAGGA-3’
	R: 5’-ACCAGGTTGTACTCGTCCAG-3’
MAPT	F: 5’- ACTCCAACAGCGGAAGATGT-3’
	R: 5’- GTGACCAGCAGCTTCGTCTT-3’
PEA-15	F: 5’- ACCCCTTCCTAATTGCAGCT-3’
	R: 5’-TGCTCTCTGGGCTCTGAAAA-3’
SERPINE1	F: 5’-CCGCCTCTTCCACAAATCAG-3’
	R: 5’-AATGTTGGTGAGGGCAGAGA-3’
SNAIL	F: 5’-CCCCAATCGGAAGCCTAACT-3’
	R: 5’-GACAGAGTCCCAGATGAGCA-3’
